# Electro-optical characteristics of a liquid crystal cell with graphene electrodes

**DOI:** 10.3762/bjnano.8.279

**Published:** 2017-12-28

**Authors:** Nune H Hakobyan, Hakob L Margaryan, Valeri K Abrahamyan, Vladimir M Aroutiounian, Arpi S Dilanchian Gharghani, Amalya B Kostanyan, Timothy D Wilkinson, Nelson Tabirian

**Affiliations:** 1 Center of Semiconductor Devices and Nanotechnologies, Yerevan State University, 1 Alex Manoogian Str., Yerevan 0025, Armenia; 2 Centre of Molecular Materials for Photonics and Electronics, Department of Engineering, University of Cambridge, 9 J. J. Thomson Avenue, Cambridge, CB3 0FA, UK; 3 BEAM Co., 1300 Lee Road, Orlando, FL, 32810, USA

**Keywords:** conductive layer, graphene, ITO, liquid crystal cell, optical switching time

## Abstract

In liquid crystal devices (LCDs) the indium tin oxide (ITO) films are traditionally used as transparent and conductive electrodes. However, today, due to the development of multichannel optical communication, the need for flexible LCDs and multilayer structures has grown. For this application ITO films cannot be used in principle. For this problem, graphene (an ultrathin material with unique properties, e.g., high optical transparency, chemical inertness, excellent conductivity) is an excellent candidate. In this work, the electro-optical and dynamic characteristics of a liquid crystal (LC) cell with graphene and ITO transparent conducting layers are investigated. To insure uniform thickness of the LC layer, as well as the same orientation boundary conditions, a hybrid LC cell containing graphene and ITO conductive layers has been prepared. The characteristics of LC cells with both types of conducting layers were found to be similar, indicating that graphene can be successfully used as a transparent conductive layer in LC devices.

## Introduction

In modern optical devices based on liquid crystals (LCs) the electro-optical control is realized using a transparent conductive layer, which is often a thin film of metal (e.g., Au, Ag) or indium tin oxide (ITO). However, the use of these coatings often complicates the technology because of the need for antireflection coatings, or they disturb the optical homogeneity and chemical stability [1–3]. Hence a number of technologies have been developed to obtain alternative transparent conducting materials, many of which are based on nanomaterials.

Among the various competing technologies, graphene films are showing great promise due to their high conductivity, optical transparency, chemical resistance and mechanical flexibility [4]. Many studies have been conducted on the application of graphene electrodes for light-emitting devices [5–7], photovoltaic devices [8–9], and touch screens [10], with much promise as a replacement for ITO. However, fundamental studies on the application of graphene as transparent electrodes for LC devices [11–12] have yet to be properly explored.

In this work we present the results of the investigation of the electro-optical and dynamic characteristics of LC cells employing graphene electrodes as compared with the characteristics of LC cells containing conventional ITO electrodes.

## Results and Discussion

### Synthesis of graphene films

The graphene was obtained by a chemical vapor deposition (CVD) process. Details of synthesis and extensive characterization of the CVD graphene can be found in prior works [13–14]. The monolayer graphene film was then transferred from the Cu foil to a precleaned glass substrate by using a polymer support layer of polystyrene (PS, *M*_w_ 35k, 2% w/w in toluene) and an etchant acid (FeCl_3_ aq, 0.5 M) to remove the Cu, as described elsewhere [15]. This was followed by a wash in a warm ethyl acetate bath to dissolve the supporting polymer layer.

### Device fabrication

**Figure 1:**
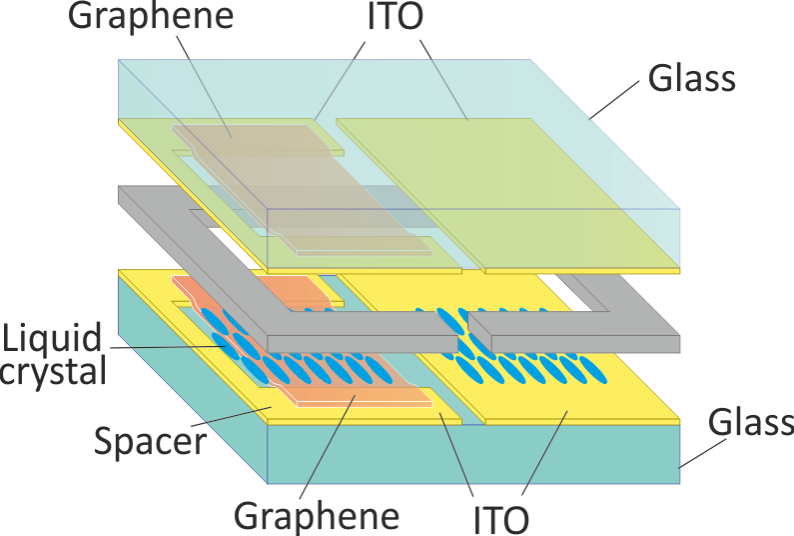
A schematic of the graphene–ITO hybrid liquid crystal cell.

Figure 1 presents the LC cell design on glass substrates. To insure the same processing conditions, the same LC layer thickness, and the same orientation boundary conditions, two structures were made in one device, each using different transparent conductive layers. Commercially available 15–20 Ohm/sq ITO coated glass substrates with 0.7 mm thickness were used as a base of the structure, which was patterned to develop two divisions in each device. The left side of the device was developed to create a graphene–graphene structure together with the right side an ITO–ITO structure, as shown in Figure 1.

**Figure 2:**
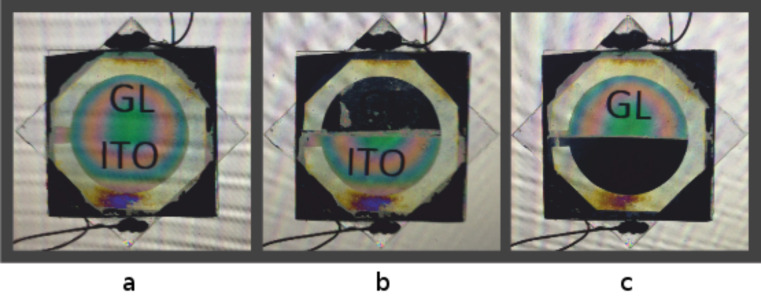
Images of the graphene–ITO hybrid liquid crystal (LC) cell between crossed polarizers: voltage not applied (a), peak-to-peak voltage *V*_pp_ = 40 V applied to LC cell with graphene electrodes (b), and with ITO electrodes (c).

To avoid inhomogeneity caused by the mechanical treatment of the substrates (rubbing), the orientation of LC molecules was realized optically (photo-orientation) [16] by irradiation of a photo-orienting polymer with linearly polarized radiation from a He–Cd laser at a wavelength of 325 nm. 6CHBT nematic LC (Δ*n* = 0.15) was filled by capillary action, and the thickness of the LC layer (20 μm) was set by spacers under planar orientation. Images of the fabricated cell, obtained between crossed polarizers when voltage is applied to different areas, are shown in Figure 2.

### Measurements

All measurements were carried out on both sides (i.e., the graphene–graphene and ITO–ITO sides) of the hybrid LC cell.

**Figure 3:**
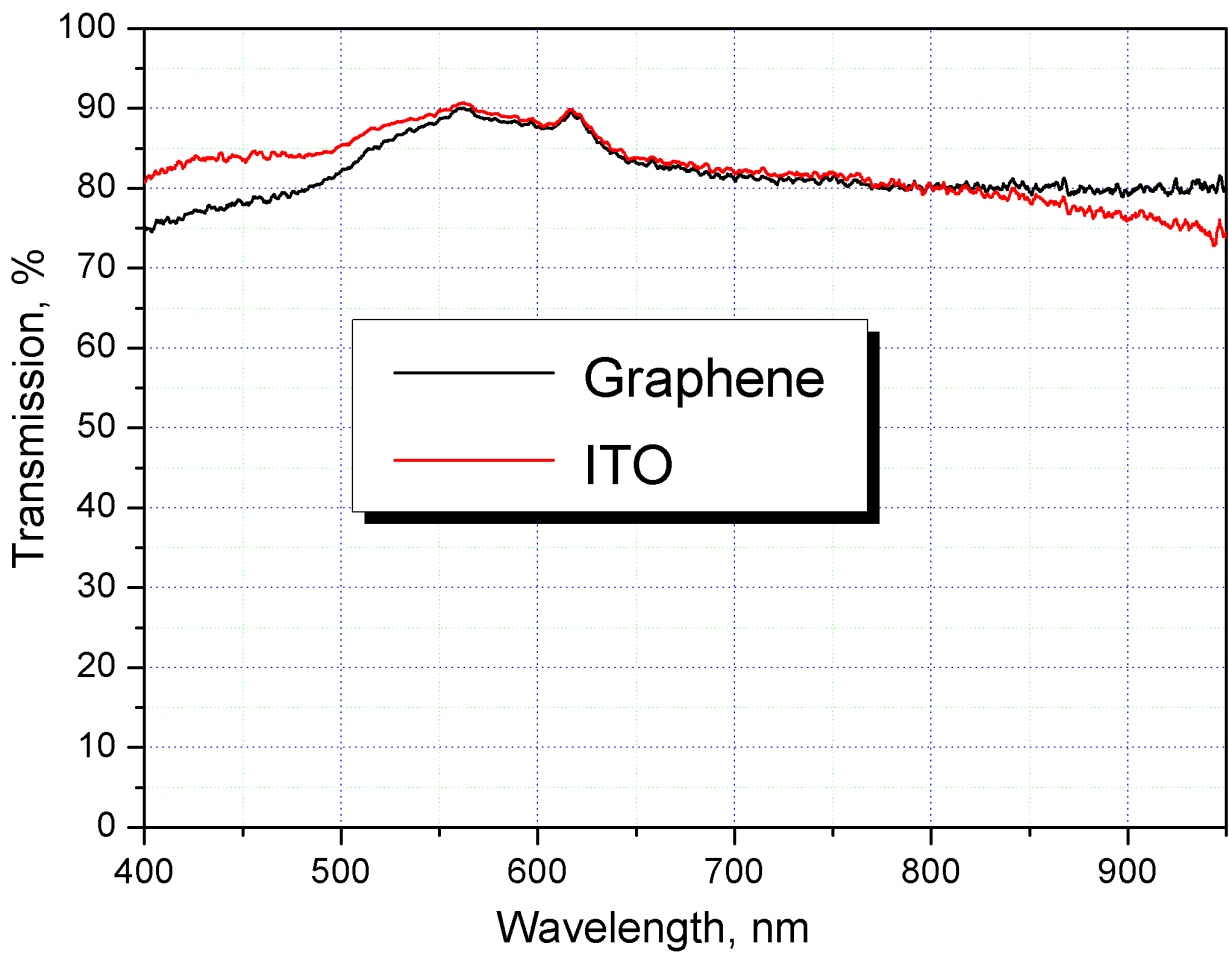
The transmission spectra of graphene and indium tin oxide sections of the hybrid liquid crystal cell.

The transmission spectra of the both sections were measured in the visible range (Figure 3). As can be seen from the figure, the cell with ITO electrodes is more transparent for the 400–500 nm wavelengths, and the cell with graphene electrodes is more transparent for wavelengths exceeding 800 nm. The transparency is the same for the 550–800 nm range.

**Figure 4:**
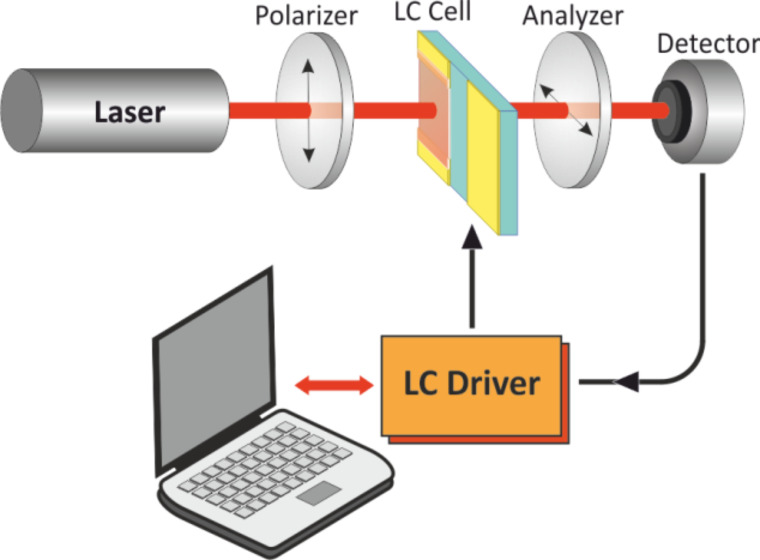
Schematic of the liquid crystal characterization experiment.

The dynamic characteristics of both sections were investigated. A schematic of the experiment is presented in Figure 4. Measurements were carried out with a specially designed driver which allows selection of the form of applied voltage, including the transient nematic effect (TNE [17]). The driver allows a smooth change of the amplitude of the control voltage with a selected rate within the given range and also registers the signal from the output of a photodetector.

**Figure 5:**
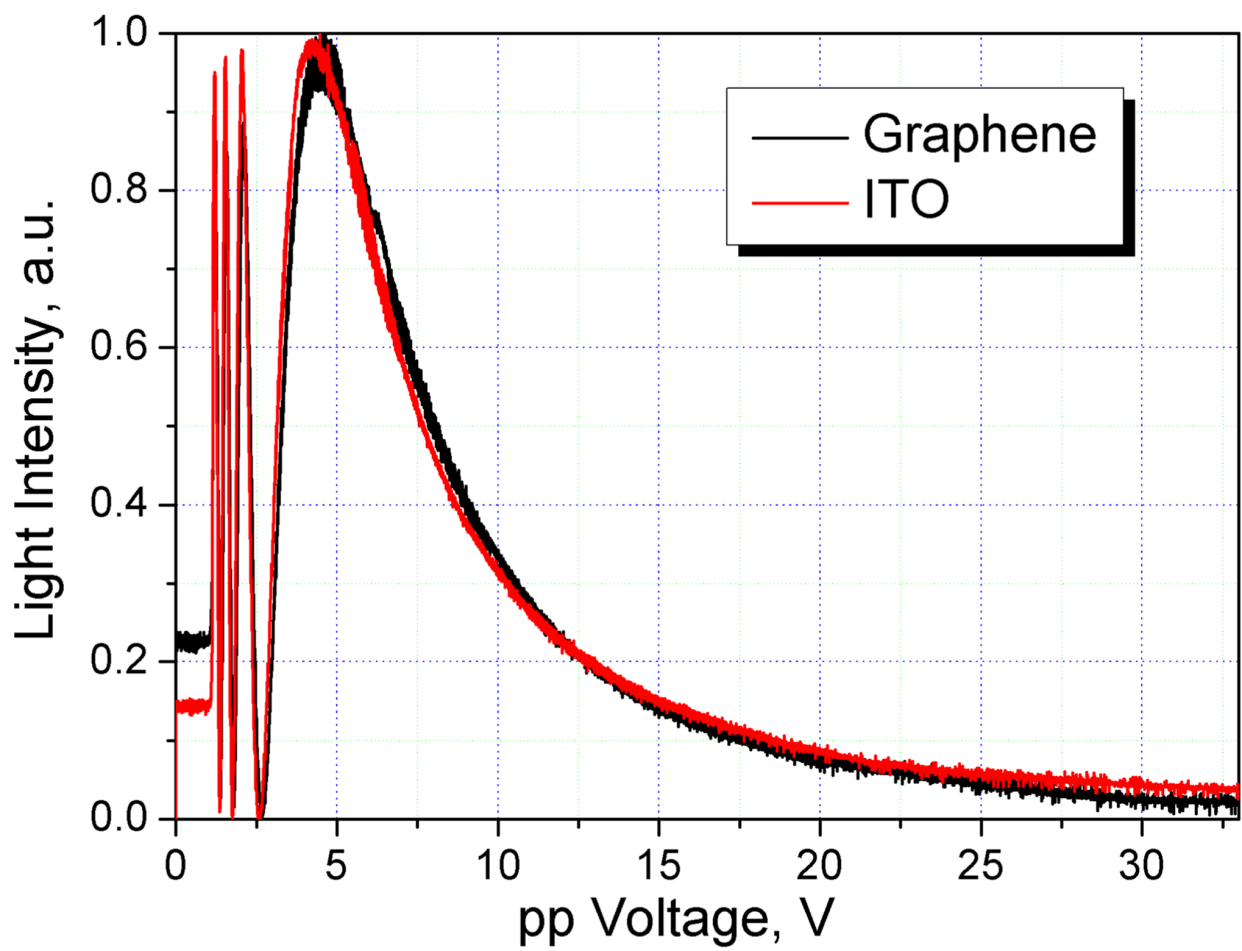
Light intensity vs peak-to-peak (pp) voltage applied, passing through the liquid crystal cell with ITO and graphene layers.

When the He–Ne laser beam (632 nm) propagates through the LC cell placed between crossed polarizers (so that the direction of the polarization of the incident beam makes a 45° angle with the LC director), then the influence of an external electric field allows typical oscillations due to the reorientation of the LC to be observed (Figure 5). Hence the LC cell can operate as an electrically controlled light valve, where the intensity of the transmitted light is changed from minimum to maximum and vice versa with an associated π-phase shift.

The switching time of the light valve depends on the value of the applied voltage. In order to determine the voltage corresponding to the minimum switching time, an experiment was carried out to obtain the dependence of the light intensity (transmitted through the cell located between crossed polarizers) on the control voltage (see Figure 5). А square-wave voltage of 1 kHz frequency with the amplitude varying at a rate of 10 mV/s was applied to the cell. As presented (Figure 5), the light intensity at the output is in the form of characteristic oscillations with increasing voltage. It is important to note that both sides of the hybrid LC cell, with different conducting electrodes (i.e., graphene–graphene and ITO–ITO), show almost identical characteristics.

**Figure 6:**
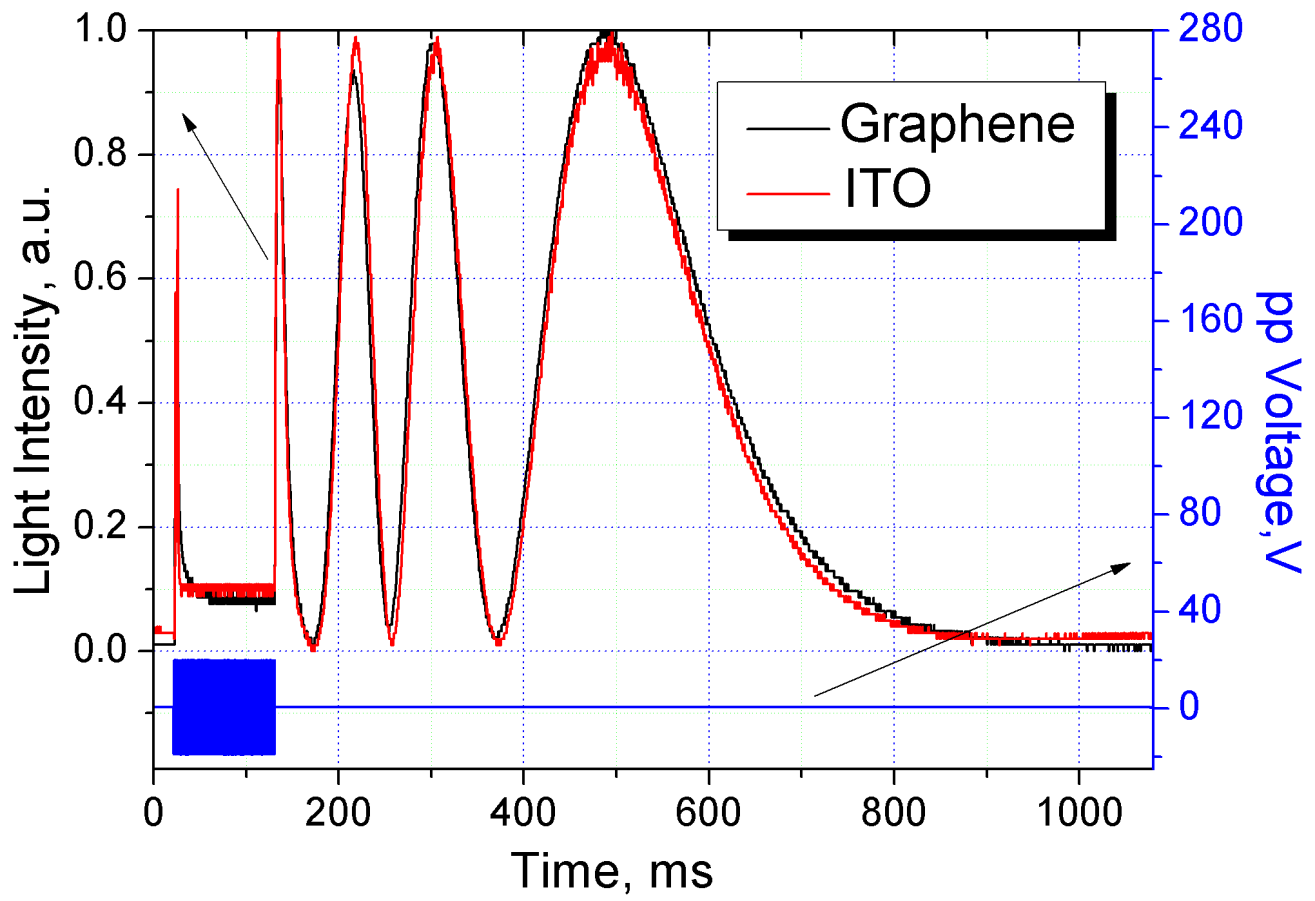
Time dependence of the intensity of light passing through the LC cells with graphene and ITO electrodes.

A similar pattern was observed during the study of the reorientation and relaxation processes under the influence of an external bipolar pulse voltage (Figure 6).

As shown, at the initial moment of the pulse action (sharp reorientation oscillation) the system tends to keep its original ordered state. Here, the translation of the highly ordered surface layer into the LC bulk occurs under the influence of elastic forces. However, later, the ordering in the bulk is lost due to the unmatched response of the LC molecules to the sharp forced impact of the electric field and the appearance of nonviscous flow (the oscillations fade away due to the strong light scattering). By the end of the pulse action, the system tends towards ordered restoration (restoration of the reorientation oscillations). Then the relaxation process takes place only under the influence of the molecular forces of elasticity. The system returns to its original oriented state, similar to relaxation after influence of an electric field. The above-mentioned feature of the reorientation process under the influence of an electric pulse, namely, a more ordered reorientation of the LC at the initial moment of its action, can be used when forming the control signal shape to improve the operating speed of LC devices.

**Figure 7:**
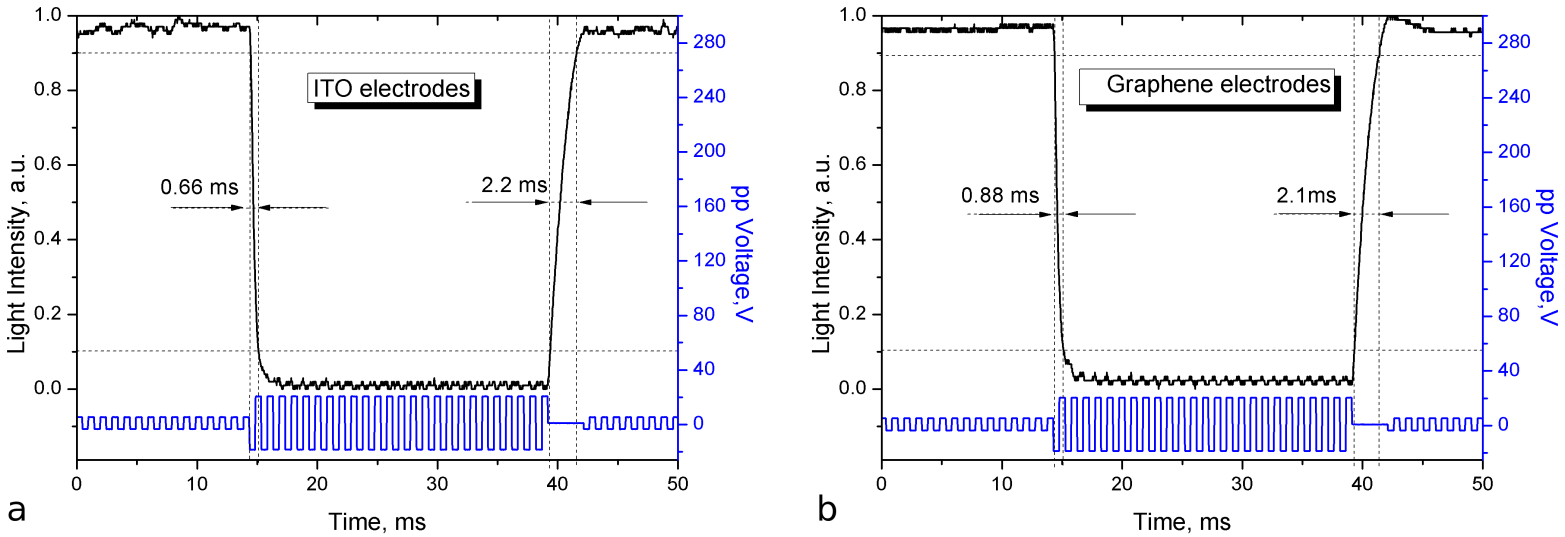
Time characteristic of liquid crystal cells with ITO (a) and graphene (b) transparent conductive layers operating as a light valve in the fast switching mode.

In general, a liquid crystal cell working as a light valve can operate in two modes: fast switching (high voltage, *V* >> *V*_th_) and slow switching (the voltage is close to the threshold, *V* < 5*V*_th_). In the fast switching mode (Figure 7), under the influence of a strong electric field, the bulk and boundary layers of the LC are involved in the reorientation process. This reduces the relaxation time due to the binding energy of the LC molecules with the surface of the substrate. Despite the short response time there are undesirable backflows, making a device operating in such mode less attractive.

In the slow switching mode, reorientation occurs in the bulk of the LC. In this case, the operation time is essentially much larger than in the previous mode due to its dependence on only the rotational viscosity and elasticity of the LC.

**Figure 8:**
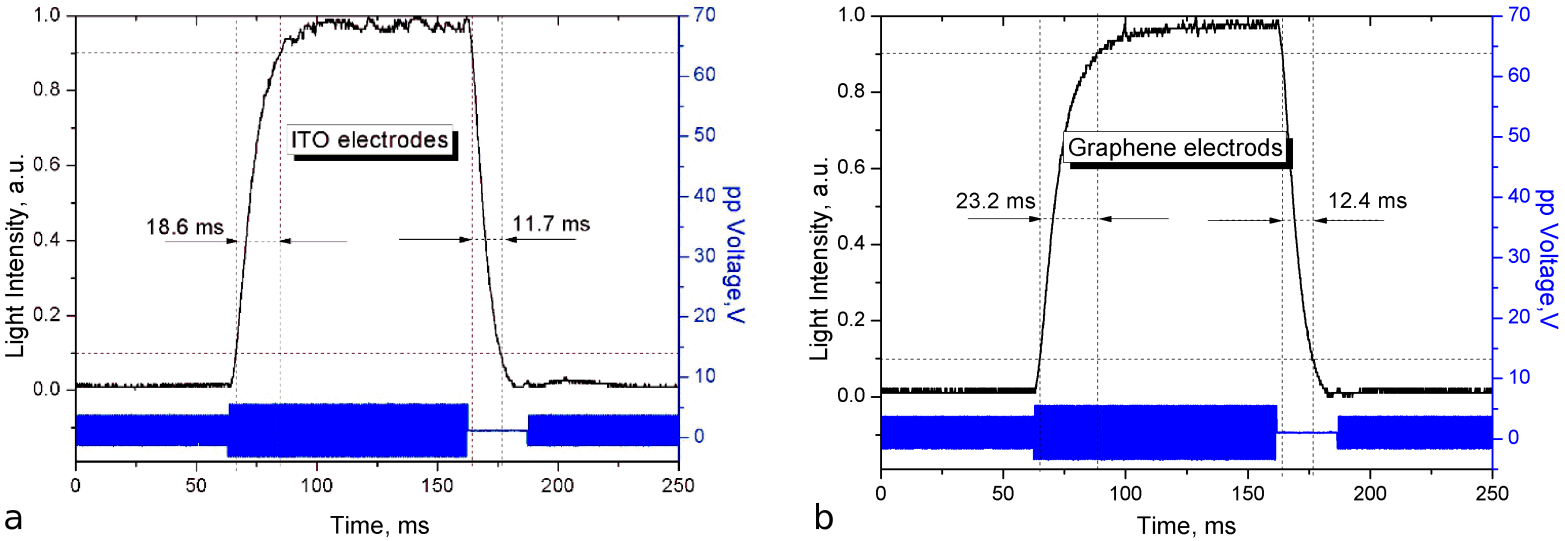
Time characteristic of liquid crystal cells with ITO (a) and graphene (b) transparent conductive layers operating as a light valve in the slow switching mode.

However, it is possible to achieve higher performance by applying a control voltage with a special shape, which is based on the transition nematic effect (TNE), when a step change in voltage leads to the switching between the quasi-steady states of the LC. Optical switching time characteristics are measured by using the TNE control signal (Figure 8).

As shown Figure 7, during the fast switching mode, the reorientation times for LC cells with ITO and graphene conducting layers are 0.66 ms and 0.88 ms, respectively, and the relaxation times are 2.2 ms (ITO) and 2.1 ms (graphene). In the slow switching mode (Figure 8) the switching times for tje LC cell with a graphene conducting layer (23.2 ms – reorientation, 12.4 ms – relaxation) are slightly higher than for the LC cell with ITO (18.6 ms – reorientation, 11.7 ms – relaxation).

## Conclusion

Hybrid graphene–ITO nematic LC devices have been investigated to characterize the electronic properties of graphene. The optical switching time characteristics of LC cells with graphene are slightly worse than those of cells with ITO. But compared to the traditional ITO, graphene has a number of advantages such as better mechanical strength, chemical resistance, the possibility to transfer onto any surface including flexible structures as well as making multilayer LC structures possible. These outstanding properties of graphene make it suitable for successful use as a transparent conductive layer in LC devices.
